# Perovskite photodetector-based single pixel color camera for artificial vision

**DOI:** 10.1038/s41377-023-01127-0

**Published:** 2023-03-22

**Authors:** Chaohao Chen, Ziyuan Li, Lan Fu

**Affiliations:** 1 School of Electrical and Data Engineering, Faculty of Engineering and Information Technology, The University of Technology Sydney, Sydney, NSW 2007 Australia; 2 Australian Research Council Centre of Excellence for Transformative Meta-Optical Systems, Department of Electronic Materials Engineering, Research School of Physics, The Australian National University, Canberra, ACT 2600 Australia; 3 MoE Key Lab of Photoelectronic Imaging Technology and System, School of Optics and Photonics, Beijing Institute of Technology, Beijing, 100081 China

**Keywords:** Optical materials and structures, Electronics, photonics and device physics

## Abstract

Narrowband red, green, blue self-filtering perovskite photodetectors and a broadband white photodetector are incorporated into a single pixel imaging camera to mimic the long-, medium-, and short-wavelength cone cells and rod cells in human visual system, leading to the demonstration of high-resolution color images in diffuse mode.

In the past decade, there has been a fast development in single pixel imaging, a powerful technique emerging as a promising alternative to multipixel image sensors, such as the silicon focal plane arrays (FPAs) featuring millions of pixels. By using a single-pixel detector, in combination with different modulation technologies and sampling schemes, single-pixel imaging has shown great advantages^1,2^ in terms of more flexibility in the choice of single photodetector (PD) devices at wavelengths across the whole electromagnetic spectrum, higher sensitivity and faster speed, precise timing resolution, reduced data storage and data transfer requirements and system costs. Indeed, since the proposal of the original idea of “Dual Photography” by ref. ^3^ in 2005 and the first demonstration of single-pixel imaging via compressive sampling by ref. ^4^ in 2008, a variety of single-pixel camera architectures^1^ have been demonstrated showing great potential for a wide range of applications. In particular, there are lots of research interests in developing infrared and THz single-pixel imagers (where focal plane detector arrays are unavailable or extremely expensive), and time-resolved imaging for 3D imaging and LiDAR applications^2^.

Conventional charge-coupled device (CCD) and complementary-metal-oxide-semiconductor (CMOS) image sensors are mature technologies that offer high resolution, fast imaging, and smart functionalities at relatively low cost, satisfying many applications in the visible wavelengths. However, for such point-to-point imaging technique, bulky optical components including lenses and bandpass filters are often requested to achieve color recognition. This, together with the inherent limitations in material properties of silicon, such as fixed bandgap and poor mechanical properties, making them less ideal for applications in artificial vision. Recently, in parallel to their significant progress in the field of photovoltaics, organic-inorganic halide perovskite materials and nanostructures have also attracted much interest for applications in color imaging and bionic visual construction^5–7^, owing to their large absorption coefficient, high mobility, low binding energy, and flexibility in adjusting chemical properties and thus bandgap energy. However, so far the reported perovskite imaging systems normally require complex manufacturing procedures and operation in the monochrome transmission mode under direct illumination due to low signal responses, making it challenging to match the performance and functionalities to the human eyesight. There are still urgent needs to develop new photodetector materials and structures, as well as imaging technologies and strategies to achieve high performance artificial vision systems.

Now, writing in this issue of Light: Science & Applications, Liu et al. at the Jinan University, China report a new perovskite photodetector-based single pixel imaging system^8^. By mimicking the long-, medium-, and short-wavelength cone cells and rod cell of human vision system using a set of red (R), green (G), and blue (B) self-filtering narrowband perovskite PDs, and a broadband white (W) PD, high-resolution (up to 256 × 256 pixels) single-pixel color imaging in diffusion mode has been achieved.

In the work presented here, the researchers propose filter-free integrated imaging PDs with highly selective responses to different wavelengths, similar to cones in the retina in the human visual system^8^. As the bandgap of metal halide perovskites can be easily adjusted by changing the composition of the halogen element (I, Br, Cl)^9^, the research team has developed a self-filtering strategy to achieve multi-color narrowband perovskite PDs. As schematically illustrated in Fig. 1, the perovskite PDs consists of two spin-coated perovskite layers, one as a perovskite filter layer (PFL), with shorter wavelength absorption against the other perovskite photodetector layer (PPL) with longer wavelength absorption. With the proper bandgap matching and thus absorption spectra overlapping, the detector is able to generate narrowband photoresponse to red, green and blue color, respectively. The PPLs are incorporated between a SnO_2_ electron transport layer and an Au electrode to form a SnO_2_/perovskite heterojunction, facilitating efficient photocarrier collection with reduced recombination, fast response time, and zero-bias operation.

**Fig. 1:**
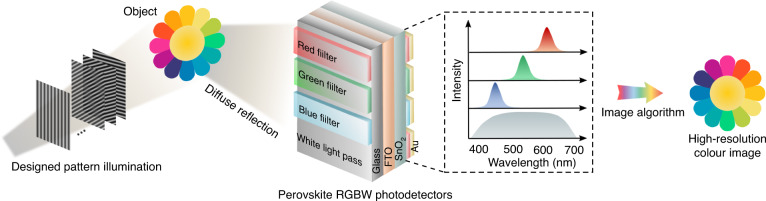
A human vision inspired perovskite-based color imaging system. Schematic of the human vision inspired perovskite-based color imaging system consisting of predesigned pattern illumination, RGBW perovskite PDs, and Fourier imaging algorithm

Furthermore, to mimic rod cells which have higher sensitivity than the cone cells, a red perovskite PD (MAPbI_2.1_Br_0.9_) without a filter layer was also fabricated next to the RGB PDs, as the white photodetector. Similar to the performance of the rod cells, this white PD exhibits a broader spectral response and better photodetection performance than the RGB PDs. Such a design forms a key element to enhance the imaging capability of the perovskite-based color camera under weak light environments.

Based on above, Liu et al. demonstrated the high-resolution perovskite PD-based color camera based on the single pixel imaging technique, as shown in Fig. 1, using predesigned pattern illumination and subsequent image reconstruction algorithm. The red, green, and blue PDs were adopted to provide the monochromatic images, respectively. Combining information from the monochromatic images into the color image enables the generation of a detailed image when the RGB PDs were exposed to adequate light (>20 μW/cm^2^); while with the assistance of the white PD and color/white image fusion using a simple image overlay method, color imaging capability was further demonstrated under weak illumination intensity (to around 5.4 μW/cm^2^).

This article provides a new perspective for exploration of new materials and strategies for the development of next generation high performance imaging camera technology. By adopting this approach, with complementary material systems and novel structures, such as III-V semiconductors^10^, 2-D materials^11^, and metasurfaces^12^, we are a step closer to mimicking of the sophisticated biological eyes that are not only sensitive to the visible light, but also to the infrared, and THz wavelengths, with associated polarization and phase information.
